# Novel therapeutic targets: bifidobacterium-mediated urea cycle regulation in colorectal cancer

**DOI:** 10.1007/s10565-024-09889-y

**Published:** 2024-08-03

**Authors:** Xusheng Nie, Tingting Zhang, Xiumei Huang, Chongqi Gu, Wei Zuo, Li-Juan Fu, Yiping Dong, Hao Liu

**Affiliations:** 1 Department of Gastroenterology, Yunyang County People’s Hospital, Chongqing, 404599 China; 2 Department of Pediatrics, Rongchang District People’s Hospital, Chongqing, 402460 China; 3 Department of Digestion, Rongchang District People’s Hospital of Chongqing, No.3, North Guangchang Road, Changyuan Street, Rongchang District, Chongqing, 402460 China; 4 Department of Herbal Medicine, School of Traditional Chinese Medicine, Chongqing Medical University, Chongqing, 400016 China; 5 Department of Pharmacology, Academician Workstation, Changsha Medical University, Changsha, 410219 China; 6 Department of Digital Medicine, Department of Bioengineering and Imaging, Army Medical University, Chongqing, 400038 China

**Keywords:** Bifidobacterium Impact, Colorectal Cancer Metabolic Regulation, Urea Cycle Modulation, Ammonia Metabolic Control, ALB Gene Influence, Colorectal Cancer Microbiota Interaction

## Abstract

**Background and purpose:**

Colorectal cancer (CRC) is a widespread malignancy with a complex and not entirely elucidated pathogenesis. This study aims to explore the role of Bifidobacterium in the urea cycle (UC) and its influence on the progression of CRC, a topic not extensively studied previously.

**Experimental approach:**

Utilizing both bioinformatics and experimental methodologies, this research involved analyzing bacterial abundance in CRC patients in comparison to healthy individuals. The study particularly focused on the abundance of BA. Additionally, transcriptomic data analysis and cellular experiments were conducted to investigate the impact of Bifidobacterium on ammonia metabolism and mitochondrial function, specifically examining its regulation of the key UC gene, ALB.

**Key results:**

The analysis revealed a significant decrease in Bifidobacterium abundance in CRC patients. Furthermore, Bifidobacterium was found to suppress ammonia metabolism and induce mitochondrial dysfunction through the regulation of the ALB gene, which is essential in the context of UC. These impacts contributed to the suppression of CRC cell proliferation, a finding corroborated by animal experimental results.

**Conclusions and implications:**

This study elucidates the molecular mechanism by which Bifidobacterium impacts CRC progression, highlighting its role in regulating key metabolic pathways. These findings provide potential targets for novel therapeutic strategies in CRC treatment, emphasizing the importance of microbiota in cancer progression.

**Supplementary Information:**

The online version contains supplementary material available at 10.1007/s10565-024-09889-y.

## Introduction

Approximately 10% of diagnosed cancers worldwide are attributed to colorectal cancer (CRC), resulting in nearly 700,000 deaths annually, making it the third most fatal cancer worldwide (Chen et al. 2020; Jiang et al. 2021; Zhao et al. 2023). Evidence from twin and familial studies suggests a heritability rate for CRC of only 12%-35%, emphasizing the significance of environmental factors in CRC development. Particularly, Western diet and lifestyle, modulated by the microbiome, are linked to CRC, posing a significant global health concern (Liu et al. 2023). While around 25–50% of patients are diagnosed early, many eventually progress to metastatic disease (Bray et al. 2020). Despite advancements in screening and treatment modalities, CRC remains curable only in its early stages. The age-standardized 5-year survival rate in South Korea ranges from 70% to 60–69% in the United States and Europe, dropping to less than 50% in Russia and India, notably with the 5-year survival rate of individuals with stage IV UICC cancer patients remaining below 20% (Montalban-Arques and Scharl 2019). Current treatments, such as immune checkpoint inhibitors (ICI), though bringing progress, benefit less than one-third of patients (Allemani et al. 2018), highlighting the urgent need for research into new therapeutic strategies. The pathogenesis of CRC is complex, influenced by both genetic factors and the environment, including the gut microbiota (Biller and Schrag 2021; Li et al. 2021a; Baidoun et al. 2021). Recent studies indicate that the gut microbiota, as a vital microbial community within the human body, is closely associated with the occurrence and progression of CRC (Barber et al. 2020; Kitamoto et al. 2020; Sui et al. 2020).

Human metabolism and immune responses are significantly impacted by the gut microbiota, which is subject to influences including age, diet, and health status (O'Keefe 2008). The progression of CRC is intricately connected to genetics, epigenetic variations, inflammation, and microbial exposure (Dejea et al. 2014; Mima et al. 2015; Siegel et al. 2020). Specific gut microbiota, such as Fusobacterium, toxin-producing Bacteroides fragilis, and Escherichia coli, are associated with the occurrence of colorectal adenomas and cancer due to their DNA-damaging effects and impact on immunity and gut barrier function (Dejea et al. 2018; Kostic et al. 2013; Tilg et al. 2018). Furthermore, studies have shown that supplementing the gut microbiota with strains like Clostridia, Lactobacillus johnsonii, and Bifidobacterium adolescentis can enhance the efficacy of platinum-based chemotherapy, cyclophosphamide, and immunotherapy by boosting anti-tumor immune responses (Daillère et al. 2016; Iida et al. 2013; Sivan et al. 2015). Despite the attention to their promoting role in cancer therapy, the specific pathways or molecular mechanisms through which they influence CRC progression remain unclear. Therefore, in-depth investigation of the gut microbiota's mechanisms in CRC is of significant importance for unraveling the pathophysiology of CRC and developing novel preventive and therapeutic strategies (Chiu et al. 2021; Xie et al. 2020; Ma et al. 2022).

Bifidobacterium, a prevalent probiotic within the gastrointestinal tract, is essential for regulating the gut microbiota and improving human health (Zaib et al. 2024). Numerous studies have documented the dominant bacterial community in the early gut microbiota—BA—which, by regulating immune responses and maintaining gut barrier function, lowers the risk of intestinal infections, including necrotizing enterocolitis in neonates (Patole et al. 2016; Jacobs et al. 2013). While the promoting role of early-life gut microbiota in cancer therapy has garnered attention, the specific pathways and molecular mechanisms through which they affect the progression of CRC remain unclear. The regulatory involvement of Bifidobacterium in the development and progression of CRC has been underscored in recent investigations (Chen et al. 2023a). Chen et al. (2020) identified that Bifidobacterium coordinates the cancer-associated fibroblasts expressing CD143, which regulate colorectal tumor development through GAS1 inhibition by the Wnt signaling pathway. This finding was supported by their study published with the PMID: 37,533,188. Additionally, Lin et al. (2023) revealed that Bifidobacterium induces Decorin( +) macrophages through TLR2 signaling to suppress the onset of CRC, as documented in their research (Lin et al. 2023). Despite these discoveries, the effects of Bifidobacterium on the progression of CRC and its underlying molecular mechanisms remain insufficiently investigated. The urea cycle (UC) is an essential metabolic pathway closely related to cellular ammonia metabolism and arginine synthesis (Chen et al. 2023b; Schimmel et al. 2023). Abnormal bile acid metabolism is associated with various diseases. For instance, an overabundance of Bifidobacterium was found in the gut microbiota of platinum-resistant individuals with epithelial ovarian cancer undergoing treatment, as reported in the study with PMID: 34,439,153. Additionally, the enhanced metabolism of lactate produced as a part of BF's metabolic activity can contribute to the promotion of the "Warburg effect" (i.e., lactate production through aerobic glycolysis), as discussed in the research highlighted by PMID: 31,737,570. This increase in lactate production in tumor cells is frequently associated with promoting angiogenesis, tumor growth, inflammation, metastasis, epithelial-mesenchymal transition, and immune evasion (Procaccianti et al. 2023). By integrating analysis of AS (Alternative Splicing) data and The cancer genome atlas (TCGA) database data, the research discovered that Bifidobacterium can affect the development of CRC by regulating UC metabolism, providing crucial evidence for further exploring the regulatory mechanisms of Bifidobacterium in CRC (Lv et al. 2022; Xavierselvan et al. 2020).

Abnormal metabolism of Bifidobacteriums has been implicated in various diseases, including tumors (Tsimberidou et al. 2020; Poore et al. 2020; Cui et al. 2021). Through the research, the research have identified differentially expressed genes related to UC metabolism and key factors regulating UC through BA. The discovery of these differentially expressed genes and key factors is of great significance in uncovering the specific mechanisms by which BAs regulate UC metabolism and the molecular basis of CRC development. Furthermore, the experimental results have also revealed that Bifidobacterium supplementation can inhibit the progression of UC by causing the accumulation of ammonia in the body and activating CRC cell mitochondrial autophagy, thus inhibiting CRC growth and development. This provides insights into the potential new strategy of using Bifidobacterium as a treatment for CRC.

The results of the study reveal that the supplementation of Bifidobacterium inhibited the onset of colorectal tumors. The research unveiled a notable decrease in the prevalence of Bifidobacterium among individuals with CRC. Introducing this beneficial bacterium as a supplement exhibited a substantial hindrance in the advancement of CRC. Furthermore, ALB was identified as a key gene involved in metabolic regulation in CRC patients; Bifidobacterium can suppress UC metabolism by downregulating ALB, thereby promoting ammonia accumulation, inducing mitochondrial dysfunction, and consequently impacting CRC progression. The protective effect of Bifidobacterium in CRC is emphasized by the research outcomes, offering a more reliable and effective approach for early diagnosis, prognosis assessment, and personalized treatment of CRC.

## Materials and methods

### Clinical sample acquisition

This study utilized tissue and fecal samples acquired from individuals suffering from CRC and those categorized as being in a healthy state between January 2020 and January 2022 at our hospital. The research involved 10 participants diagnosed with CRC and 10 healthy subjects. The detailed demographic information for the two groups of patients can be found in Table S1. Inclusion of all participants in the study followed the acquisition of informed consent from each individual. The age range of the patients was 43 to 65 years, averaging at 54.8 years. Similarly, the age range of the healthy individuals was 43 to 63 years, with a mean age of 51.6 years. The collected tissue samples were split into two parts, wherein one part was immediately preserved in liquid nitrogen, while the other part was immersed in 10% formalin, followed by processing and embedding in paraffin for subsequent sectioning (Chen et al. 2023a). The research has been granted ethical approval from the Clinical Ethics Review Committee at our medical institution, adhering to the guidelines set forth in the Helsinki Declaration.

### Acquiring 16S rRNA sequencing data

The study obtained 16S rRNA sequencing data for CRC research by searching the EMBL-EBI database (https://www.ebi.ac.uk/ena/browser/search) using the keyword "Colorectal cancer." The research found relevant projects and downloaded the phenotypic information for all samples from these projects (project accession: PRJEB6070). Additionally, the research accessed the NCBI Sequence Read Archive (SRA) database (https://www.ncbi.nlm.nih.gov/sra/) to retrieve the 16S rRNA sequencing data for these samples. In order to minimize sample bias and reduce computational costs, the research employed the 'sample()' function in R to randomly select sequencing data from 6 healthy individual stool samples and 6 CRC patient stool samples included in this study (Clarridge 2004).

### Microbial relative abundance analysis

The research employed multiQC and kneaddata tools for the evaluation of the samples. multiQC was used to perform sequence quality control, while kneaddata aided in the removal of host and contaminant sequences. For the construction of the microbial species tree and the annotation of differences, the study implemented GraPhlAn, which provided us with the relative abundance of microbial classifications. The assessment of species complexity diversity within the samples was conducted using the Simpson index for Alpha diversity analysis, and the analysis of beta diversity was executed through Principal Coordinates Analysis (PCoA). Bacterial abundance and diversity were compared using the Wilcoxon rank-sum test and Welch's t-test. Moreover, the R package edgeR was utilized to compute inter-group abundance differences, and the study visualized the results through volcano plots and Manhattan plots (Kang et al. 2021).

### Microbial functional composition

The FAPROTAX dataset and associated software provide an artificially constructed database of prokaryotic functional groups for predicting the main ecological functions of microbial communities. The investigation employed the QIIME data and converted it into an R package for analysis, conducting community phylogenetic investigations using PICRUSt. The Kyoto Encyclopedia of Genes and Genomes (KEGG) was used to predict the metagenomic pathways for each primer set (Kanehisa et al. 2016; Douglas et al. 2020). To perform statistical analysis and visualization of the unstratified results, the study utilized the software STAMP (v2.1.3). The comparison of functional composition variances utilized Welch's t-test (Parks et al. 2014).

### Obtaining and cultivating bacterial strains

The Bifidobacterium adolescentis (B.a) strain of bacteria was obtained from the American Type Culture Collection (ATCC15703, ATCC, USA). These bacteria underwent a cultivation for 48 h in an improved anaerobic enrichment medium under the conditions of 10% H_2_, 10% CO_2_, and 80% N_2_. E. coli DH5a strain (No.9057, Takara) was incubated in Luria–Bertani medium (A507002, Sangon Biotech, Shanghai, China) at 37 °C to function as a negative control for non-pathogenic commensal gut bacteria. When the optical density (OD) of the B.a strain reached 1.0 at 600 nm, the bacteria were recovered by centrifugation at 1000 × g for 5 min at 4 °C. The bacterial sediments were subsequently rinsed two times with sterile anaerobic phosphate-buffered saline (PBS), reconstituted in strict anaerobic conditions, and finally adjusted to a density of 1 × 10^9^ CFU/300 μL.

### DNA extraction and quantification of bacterial DNA

The QIAGEN Stool Kit (51,604, QIAGEN, Germany) was utilized for the extraction of bacterial DNA from human fecal specimens. Subsequently, the study extracted bacterial genomic DNA (gDNA) from human tissue following the manufacturer's protocol using the QIAGEN DNA Mini Kit (56,304, QIAGEN). For quantification, the study performed real-time PCR (qRT-PCR) via the Roche LightCycler® 480 system (Rotor gene 6000 Software, Sydney, Australia) targeting the B.a gene, universal bacterial 16S gene, and PGT gene. Every reaction was carried out three times employing SYBR Green Master Mix (RR820A, Takara) as the fluorescent probe, with a template gDNA concentration of 100 ng. Relative abundance was analyzed applying the -ΔCt approach. The universal bacterial 16S gene served as the internal reference for fecal samples, and the PGT gene served as the internal control for tissue samples. Table S2 outlines the specific primers that were used.

### Preparation of CRC model in mice

Forty-eight 8-week-old C57BL/6N mice (Catalog No. 213) were procured from Beijing Vital River Laboratory Animal Technology Co., Ltd. (Beijing, China). The mice were accommodated in conventional cages and maintained at a constant room temperature (23 ± 1 °C) under a 12-h light/dark regimen. Food and water were available to them at all times without any restrictions. Prior to the experiment, the mice underwent a one-week acclimation period. This study protocol and the use of animals were authorized by the Ethics Committee of our institution.

Six experimental groups were formed for the mice: the Control group (fed with PBS), the E.coli group (fed with Escherichia coli), the B.a group (fed with B.a), the B.a + PBS group (fed with B.a and injected with PBS), the B.a + rALB group (fed with B.a and injected with recombinant albumin), the B.a group (fed with B.a for sequencing), and the Control group (fed with PBS for sequencing) (Chen et al. 2023a).

Bacterial feeding treatment was performed subsequent to treatment with streptomycin. Mice were orally gavaged with 1 × 10^9^ colony-forming units (CFU) of B.a, E.coli, or PBS in the identical quantity thrice weekly for a duration of 120 days (Chen et al. 2023a).

ALB overexpression was achieved by intraperitoneal injection of recombinant albumin at a concentration of 1.2 mg/kg. The control group received the same dosage of PBS. Injections were performed every three days from the beginning of the experiment until its completion (Mugaka et al. 2021; Tyagi et al. 2021).

Weekly body weight assessments of mice were carried out during the experiment, with the observation of anal prolapse in the last month. Following a period of four months, fasting was implemented before the collection of both colon and tumor tissues for subsequent experiments. Colon tissue photos were taken, and the number and size of tumors were evaluated. Tumor size (diameter) was categorized as < 1 mm, 1–2 mm, 2–3 mm, or > 3 mm. Tumor burden was defined as the total sum of tumor diameters per mouse. Tumor tissue photos were taken, and their weight was documented (Chen et al. 2023a).

### RNA extraction and sequencing

Colonic tissue samples from four mice in the B.a group and four mice in the Control (PBS-fed) group were collected for total RNA extraction using Trizol reagent (Invitrogen, 15,596,026, Carlsbad, CA, USA). Assessment of RNA samples' concentration and purity was measured applying the NanoDrop 2000 spectrophotometer (NanoDrop Technologies, 1011U, Wilmington, DE, USA). Only total RNA samples adhering to the specified criteria were included in the subsequent investigations: RNA Integrity Number (RIN) ≥ 7.0 and 28S:18S ratio ≥ 1.5.

CapitalBio Technology (Beijing, China) performed the generation and sequencing of the libraries. Five micrograms of RNA were used for each sample. Briefly, the Ribo-Zero™ Magnetic Kit (Epicentre Technologies, MRZE706, Madison, WI, USA) was employed to deplete ribosomal RNA (rRNA) from total RNA. The NEB Next Ultra RNA Library Prep Kit (New England Biolabs, #E7775, Ipswich, MA, USA) was utilized to construct libraries for Illumina sequencing. The RNA fragments were fragmented together with products of around 300 base pairs (bp) in length in the first strand synthesis reaction buffer (5 ×) from NEB Next. The first strand cDNA was generated through the utilization of reverse transcriptase primers and random primers, followed by a second strand synthesis reaction in the presence of dUTP Mix (10 ×) in the buffer. The cDNA fragments underwent end repair, including the implementation of polyA tails and ligation of sequencing adapters. After the ligation of Illumina sequencing adapters, the second strand of cDNA was treated with the USER enzyme (New England Biolabs, #M5508) for the creation of strand-specific libraries. Amplification and purification were carried out on the library DNA before enrichment through PCR. Finally, library identification was carried out via the Agilent 2100 Bioanalyzer, and library quantification was performed applying the KAPA Library Quantification Kit (KAPA Biosystems, KK4844). Ultimately, paired-end sequencing was conducted using the NextSeq CN500 (Illumina) sequencer (Reuter et al. 2015).

### Quality control of sequencing data and alignment to the reference genome

The original paired-end reads were subjected to quality assessment utilizing FastQC software v0.11.8. Subsequently, Cutadapt software 1.18 was utilized to preprocess the raw data, removing Illumina sequencing adapters and poly(A) tails. Then, a Perl script was employed to discard reads with an N content exceeding 5%. Following this, using the FASTX Toolkit software version 0.0.13, reads meeting a base quality threshold higher than 20 were extracted, comprising 70% of the reads. Pair-end sequences underwent repair through the application of BBMap software. The last step involved aligning the refined and top-notch read fragments to the mouse reference genome utilizing hisat2 software (version 0.7.12) (Reuter et al. 2015).

### Differential gene analysis

For the analysis of differential gene expression in the sequencing data, the "limma" package in R software was employed (Ritchie et al. 2015). In the analysis for identifying differentially expressed genes related to ulcerative colitis (UC), genes with │log FC│ > 2 and a p-value < 0.05 were defined as differentially expressed. For identifying differentially expressed genes related to Bifidobacterium, the cutoff criteria used were │log FC│ > 0.5 and a p-value < 0.05.

In order to visualize the results of the differential analysis, the "ggplot2" package in R software was employed to create volcano plots (Tan et al. 2020). To identify the intersection of differentially expressed genes or UC-related genes, the "VennDiagram" package in R software was utilized to plot Venn diagrams (Luo and Ma 2020). Information on metabolism-related genes in UC was sourced from the GeneCard database (https://www.genecards.org/).

### Analysis of ALB protein expression in the HPA database

The investigation queried the HPA database (https://www.proteinatlas.org/) using the search term "ALB" and selected the "COLON" option in the "TISSUE" module to download immunohistochemistry images of ALB in normal colon tissues. Additionally, the study chose the "COLORECTAL CANCER" option in the "PATHOLOGY" module to download immunohistochemistry images of ALB in tumor tissues from CRC patients.

### Lentivirus infection

To investigate the overexpression of ALB in cells, the study performed a transfection experiment using lentivirus-mediated gene delivery. Initially, 5 × 10^5^ tumor cells were inserted into a 6-well plate and cultured until reaching 70–90% confluence. Subsequently, the cellular transfection was performed with a viral titer of approximately 5 × 10^6^ TU/mL, with a multiplicity of infection (MOI) of 10, along with 5 μg/mL polybrene (Merck, TR-1003, USA). The transfection was conducted for 4 h, followed by the addition of an equivalent volume of culture medium to dilute the polybrene. Subsequent to a 24-h transfection, the old medium was substituted with a new culture medium. Transfection efficiency was evaluated at 48 h post-transfection using a luciferase reporter gene, and stable cell lines were selected through 1 μg/mL puromycin (A1113803, Thermo Fisher, USA) (Jia et al. 2021; Hu et al. 2021; Li et al. 2018). Each experimental group was repeated three times. The recombinant lentivirus and plasmid constructs were kindly provided by Shanghai Biotech Co., Ltd., located in Shanghai, China.

### Cell culture

The CRC cell lines used in this study, including HCT116 (CCL-247) and SW480 (CCL-228), were obtained from the American Type Culture Collection (ATCC) and appropriately stored in our laboratory. To ensure the purity and quality of cell cultures, regular testing for mycoplasma contamination was performed using the Bitool Mycoplasma Detection Kit. The following are the specific steps involved in the cell culture process:

- Thaw the cryopreserved cells from liquid nitrogen and rapidly warm them in a water bath set at 37 °C.
- Transfer the thawed cells into a 15 mL centrifuge tube containing pre-warmed 10 mL complete DMEM (11,965,092, Gibco, USA).
- Spin the sample at 300 g for 5 min, eliminate the supernatant and incubate the cells in a 37 °C, 5% CO_2_ incubator (Heracell™ Vios 160i CR CO_2_ incubator, 51,033,770, Thermo Scientific™, Germany).
- When the cells reach 80% ~ 90% confluency, passaging is performed (Lyu et al. 2021; Zhu et al. 2021a; Zhang et al. 2019).
- During passage, wash the cells with PBS 1–2 times, then introduce the cells to an adequate trypsin–EDTA solution for detachment and continue incubating in the 37 °C incubator for 5 min.
- Inspect the cells through the lens of a microscope, and once cells start detaching, add complete DMEM to stop the digestion.
- Transfer the cell suspension to a new centrifuge tube, centrifuge at 300 g for 5 min, discard the supernatant, and then resuspend the cells in fresh, complete DMEM.
- Replace the growth medium every 2–3 days based on cell growth and changes in the color of the culture medium (Lyu et al. 2021; Zhu et al. 2021a; Zhang et al. 2019).

For the cell culture under B.a stimulation, CRC cells of interest were inoculated into 6-well plates with a concentration of 2 × 10^5^ cells per well and cultured overnight in DMEM (11,965,092, Gibco, USA). Then, the cells were treated with B.a at a 100:1 MOI, and incubated for an additional 48 h in preparation for the experiments. The control group was treated with the identical volume of PBS (Chen et al. 2023a).

The experimental groups were as follows: 1. Ctrl group: CRC cells + PBS; 2. B.a group: Co-culture of CRC cells with B.a; 3. B.a + oe-NC group: Co-culture of CRC cells transfected with control sequence and B.a; 4. B.a + oe-ALB group: Co-culture of CRC cells transfected with ALB sequence and B.a.

### Western blot

The investigation employed a protein extraction kit (Bestbio, BB3101, Shanghai, China) to extract total protein from the samples, and the determination of protein concentration was executed with the aid of a BCA assay kit (Beyotime, P0012S, Shanghai, China). A 10% SDS-PAGE gel was prepared (Beyotime, P0012A, Shanghai, China), with each well loaded with 50 μg of protein samples. A steady voltage of 80 V was applied for 2 h during the electrophoresis process, followed by increasing to 120 V. Subsequently, the gel-to-PVDF (Merck, IPVH00010, Germany) membrane transfer process was implemented for the proteins. The blocking of the PVDF membrane was accomplished through the application of 5% skim milk in TBST (Tris-buffered saline with Tween-20) at room temperature for 2 h, followed by removal of the blocking solution and washing the membrane with TBST for 10 min. Afterward, the membrane underwent incubation overnight under 4℃ conditions with the primary antibody (antibody information provided in Table S3; Thermo Fisher; Abcam). The membrane was then washed three times with TBST for 10 min each. Subsequently, the membrane was incubated with Goat anti-rabbit IgG (1:2000, Abcam, ab6721, United Kingdom) or Goat anti-mouse IgG (1:2000, Abcam, ab6789, United Kingdom) conjugated with horseradish peroxidase at ambient temperature for a duration of 1 h. Subsequent to three more TBST washes of 10 min duration per wash, the membrane was developed using an ECL reaction kit (Beyotime, P0018FS, Shanghai, China) and exposed in a dark box for visualization (Chen et al. 2022). Each sample experiment was performed in triplicate.

### RT-qPCR detection of gene expression

Total RNA was obtained by extracting from the samples using Trizol (Thermo Fisher, 16,096,020, USA) acidic reagent, and cDNA was synthesized via the reverse transcription reagent kit (Takara, RR047A, Japan). The preparation of the reaction system was carried out with the One Step TB Green® PrimeScript™ RT-PCR Kit (Takara, RR066A, Japan), and the RT-qPCR reaction was carried out in the real-time fluorescence quantitative PCR instrument (Thermo Fisher, ABI 7500, USA). The internal reference control in this study was designated as GAPDH. The PCR procedure was structured with the following parameters: initial denaturation at 95 °C for 30 s, followed by cycles of denaturation at 95 °C for 5 s, annealing at 60 °C for 30 s, repeated for 40 cycles. This was followed by an extension at 95 °C for 15 s, 60 °C for 60 s, and a final extension at 90 °C for 15 s. Amplification curves were further illustrated in the study. All RT-qPCR experiments were performed with triplicate wells. The primer sequences can be found in Table S2. The 2-^ΔΔCt^ method was used to represent the relative expression ratio of the target gene between the experimental group and the control group, where ΔΔCT = ΔCt _test_—ΔCt _control_, and ΔCt = Ct _target_—Ct _reference_. The experiments were repeated three times.

### Colony formation experiment

First, HCT116 and SW480 cells were plated in a 60 mm culture dish with a density of 1 × 10^3^ cells and treated separately with B.a for 24 h. Subsequently, fixation of colonies was carried out by exposure to 4% paraformaldehyde at room temperature for 30 min. Next, the cells were washed twice with PBS solution and stained with Wright-Gimsa staining solution (C0121, Beyotime, Shanghai, China). Then, the culture plates were visually examined, and the quantity of colonies was computed (Shen et al. 2019). Each test was iterated thrice for accuracy.

### Metabolite analysis by LC–MS

For tissue samples, the study obtained 60 mg of HCT116 xenografts and added 200 μL of pre-chilled ultrapure water to each sample for homogenization using MP. Cell samples involved seeding HCT116 cells in 6 cm culture dishes, followed by an overnight incubation. After 24 h, the cells were treated with B.a for 24 h. Subsequently, metabolites were extracted after harvesting the cells. This involved taking each sample, adding 500 μL of pre-chilled 0.3% ammonium acetate solution, washing the samples twice, and then quenching with liquid nitrogen.

Next, pre-chilled MeOH/ACN/H2O (2:2:1, v/v/v) was added to the samples, and cells were scraped off and transferred to 1.5 mL centrifuge tubes. Samples were subjected to vortexing for 30 s and sonication for 20 min while being kept in an ice bath. Protein precipitation was achieved by incubating the samples at 1 °C for 20 h, followed by centrifugation at a speed of 20,000 rpm and a temperature of 4 °C for a brief period of 4 min. The supernatant was transferred to a standard volume and dried using a vacuum concentrator.

The dried extracts were reconstituted in 100 mL of acetonitrile water (1:1, v/v) and subjected to 10 min of sonication. Subsequently, centrifugation was carried out at 15,000 rpm and 4 °C for 4 min to remove insoluble debris. Aminoacetyl derivatives were identified and quantified using liquid chromatography-tandem mass spectrometry (LC–MS/MS). A high-resolution separation of amino acids was performed using an Agilent 150 Infinity liquid chromatography system incorporating a Zic-HILIC column (1290.3 μm, 5.2 mm × 1 mm). The injected volume was set at 2 μL (Lin et al. 2017).

The mobile phase was composed of an eluent A, which was a wash solution composed of 25 mM HCOONH (containing 0.08% HCOOH), and an eluent B, which was an elution solution composed of acetonitrile containing 0.1% HCOOH. Separation was carried out using a linear gradient of eluent B (0–12 min, 90% B to 70% B; 12–18 min, 70% B to 50% B; 18–25 min, 50% B to 40% B; 25–30 min, 40% B) at a flow rate of 250 μL/min. A constant temperature of 40 °C was maintained for the column. The acquisition of all mass spectrometry data was performed through a mass spectrometry selection detector (5500 QTRAP) coupled with electrospray ionization (ESI) source, with conditions including a source temperature of 500 °C, an ion source gas 1 (GS1) of 40, an ion source gas 2 (GS2) of 40, a curtain gas (CUR) of 30, and an ion spray voltage floating (ISVF) at 5500 V.

The positive ion ESI mass spectrum was recorded in the range of 50–2200 m/z. All ion pairs for aminoacetyl derivatives were detected using the MRM mode. For metabolite identification, peak areas and retention times were extracted with the assistance of Multiquant software. Retention times were calibrated using aminoacetyl derivative standards, and retention times and the extraction of peak areas for metabolite identification was conducted through the utilization of Multiquant software. Finally, the creation of boxplots to visualize amino acid data was achieved using R packages (Zhang et al. 1990).

### Histological staining

Initially, tissue samples were acquired and subjected to fixation. Subsequently, wax blocks were sectioned, followed by dewaxing using xylene and dehydration using a solution series of 100% ethanol, 95% ethanol, and 70% ethanol. The sections were then rinsed with water or immersed in it. Next, the prepared sections were stained with hematoxylin (H8070, Solarbio, Beijing, China) for 5–10 min at ambient temperature. Subsequently, the segments were rinsed with purified water, dehydrated in 95% ethanol, and finally immersed in an eosin staining solution (G1100, Solarbio, Beijing, China) for an additional 5–10 min of staining. Subsequently, routine steps for dehydration, transparency, and mounting were performed (Bian et al. 2021).

### Oxidative stress levels

The assessment of malondialdehyde (MDA) levels was performed using the thiobarbituric acid reactive substances assay kit (S0131S, Beyotime, China). This assay can be performed using either cell or tissue samples. Specifically, 0.1 mL of the sample was added to 0.2 mM MDA detection working solution. After thorough mixing, the sample was subjected to a temperature of 100 °C or in a boiling water bath for 15 min. Subsequently, cooling of the sample to room temperature was achieved by immersion in a water bath. Following this, the sample was centrifuged at 1000 g for 10 min at room temperature. Then, 200 μL of the supernatant was collected and added to a 96-well plate. Quantification of MDA content was carried out by measuring the absorbance at 532 nm.

Moreover, the levels of superoxide dismutase (SOD) were evaluated using different assay kits from Beyotime (S0088, China). Determination of SOD content was achieved through absorbance measurement at 560 nm (Zhu et al. 2021b).

### Flow cytometry method for detecting ROS levels

As mentioned earlier, intracellular levels of reactive oxygen species (ROS) were examined via flow cytometry (Shan et al. 2015). In brief, seeding of CRC cells was performed in 60 mm cell culture plates, followed by an overnight incubation at 37 °C under 5% CO_2_. Subsequently, experiments were conducted following the instructions of the Reactive Oxygen Species Assay Kit (S0033S, Shanghai, China) from Beyotime Biotechnology. Initially, the cells underwent two rounds of washing with PBS., and then 2,7-dichlorodihydrofluorescein diacetate (DCFH-DA) fluorescent probe (10 mol/L) was added to the supernatant, with a volume of 190 mL. The samples were then incubated in the dark at 37 °C for 30 min. The presence of fluorescent dichlorofluorescein (DCF) indicated the production of ROS. Measurement of fluorescence intensity was conducted by flow cytometry after cell retrieval (Shan et al. 2022).

In order to detect ROS production in live cells, CRC cells plated on coverslips were rinsed with preheated PBS and subsequently treated with 3 μM dihydroethidium (DHE) dye from Sigma-Aldrich at 37 °C for 30 min. After washing, observation of the cells was performed through a Zeiss confocal microscope (LSM 750, Gottingen, Germany) using a 20 × objective lens. ImageJ software was used to quantify the DHE fluorescence intensity, and the average fluorescence intensity of six randomly selected fields in each trial was calculated as a multiple relative to the control group.

### EdU proliferation assay

Cell cultures were established in 24-well plates and allowed to grow overnight in controlled environments. Within 24 h after overnight culture, HCT116 and SW480 cells were treated with or without NH4CI (5 mM). To evaluate cell proliferation, the BeyoClick™ EdU Cell Proliferation Assay Kit (C10337, Thermo Fisher, USA) was used to label EdU with Alexa Fluor 488. Cell exposure to 10 μM EdU lasted for a period of 2 h, followed by fixation, permeabilization, and EdU staining in compliance with the manufacturer's guidance. Hoechst 33,342 was used for nuclear staining, with a processing time of 20 min. Fluorescence microscopy was employed to calculate the ratio of cells containing EdU. Five fields were randomly selected in each experiment, and the number of EdU-positive cells (fluorescently labeled) and the total cell count (nuclei stained with Hoechst 33,342) were recorded to calculate the percentage of EdU-positive cells: (EdU-positive cell count/total cell count) × 100% (Angelova et al. 2022). The average values were obtained from three repetitions of each experiment.

### Detection of mitochondrial membrane potential (Δψm) using TMRM staining

The mitochondrial membrane potential assay kit was purchased from BioVision (catalog number HR8273). In simple terms, cells were cultured in appropriate culture plates until they reached the desired growth stage. Afterwards, the cell cultures were exposed to slices immersed in a culture medium containing 25 nM TMRM and incubated at 37 °C for 40 min. After staining, the cells underwent gentle rinsing with PBS buffer 2–3 times to remove TMRM that did not enter the cells. Employing a fluorescence microscope equipped with an excitation wavelength of 561 nm, equipped with a 40 × oil immersion objective and a long-pass filter, the fluorescence of TMRM was detected, and Z-stack images were captured to represent the Z-axis. To validate the specificity of the observed results, it is recommended to treat a portion of the cells with a known mitochondrial depolarizing agent (such as FCCP) as a control. Finally, in the Z-stack, image analysis software was utilized to measure and contrast the TMRM fluorescence intensity with the control group (Angelova et al. 2022).

### ELISA assay kit for ATP and mitochondrial complexes detection

ATP (ml095893), Complex I (ml058155), Complex II (ml058156), Complex III (ml058157), and Complex IV (ml058158) in CRC cells were purchased from ElisaBiotech. The samples were processed complying with the specifications outlined by the manufacturer and then analyzed (Yokoo et al. 2021).

### Immunohistochemical staining

Embedding and sectioning procedures were performed on mouse tumor tissues, followed by a 20-min bake at 60 °C. The next step involved immersing the sections in a xylene solution, changing the xylene twice, with each immersion lasting for 15 min. Subsequent to a 5-min immersion in absolute ethanol, the sections underwent an additional 5-min treatment with fresh absolute ethanol. Hydration was carried out by immersing the sections in 95% and 70% ethanol for 10 min each.

In order to eliminate endogenous peroxidase activity, each section was incubated with 3% H_2_O_2_ at room temperature for 10 min. Afterward, the sections were placed in a microwave oven with citrate buffer and boiled for 3 min. Antigen retrieval was achieved by incubating the sections with antigen retrieval solution for 10 min at room temperature. The sections underwent a triple wash with PBS.

Normal goat serum blocking solution (catalog number: E510009, Shanghai Bioengineering Co., Ltd.) was introduced into the sections and left to incubate at ambient temperature for 20 min. Diluted PCNA primary antibody (catalog number: 13–3900, Thermo Fisher, USA) was added and incubated at 4 °C overnight. The next day, the sections received three washes with PBS. Goat anti-mouse IgG1 secondary antibody (A10551, Thermo Fisher, USA) was applied and incubated for 30 min. Subsequent to the PBS wash, the sections were stained with the DAB staining kit (DAB150, Sigma, USA) by adding a drop of each component (A, B, and C) onto the sections and incubating for 6 min. Hematoxylin staining of the sections lasted for 30 s.

Sequential dehydration was carried out by immersing the sections in 70%, 80%, 90%, 95% ethanol, and finally absolute ethanol, with each concentration for 2 min. The sections were then immersed in xylene twice for 5 min each. The samples were mounted using neutral resin (Li et al. 2020). The sections were observed under an Olympus BX63 upright microscope. The positive protein expression areas were determined using the Aperio Scanscope System image analysis system in Vista, CA (Li et al. 2020).

### Statistical analysis

In this study, the statistical software R version 4.2.1 was used, and the code was compiled using the RStudio version 4.2.1 integrated development environment. The Perl language version 5.30.0 was utilized for processing files. Cytoscape version 3.7.2 was used for network analysis. The statistical software SPSS version 21.0 (IBM SPSS Statistics, Chicago, USA) was employed for data analysis. Descriptive statistics presented the data as mean ± standard deviation. Independent sample t-tests were used to compare between two groups. A repeated measures analysis of variance was employed to compare variations at various time intervals within each group, followed by post-hoc tests with Bonferroni correction. The statistical significance threshold was set at P < 0.05, indicating statistical significance.

## Results

### Intestinal microbiota in the development of CRC: role and differential studies

**Fig. 1:**
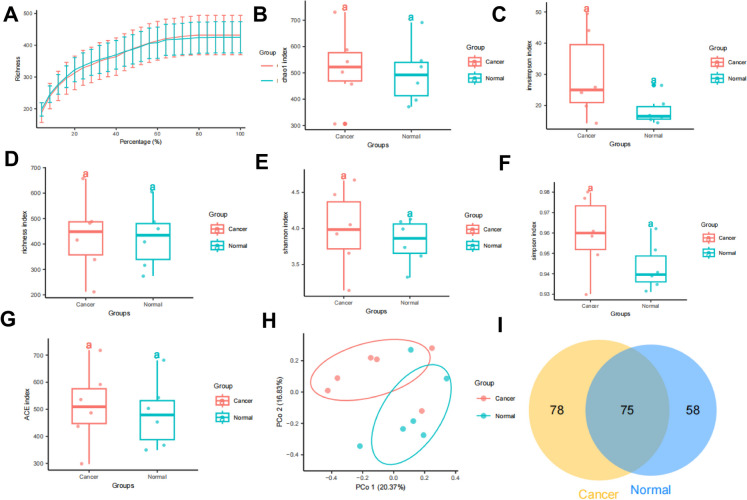
Analysis of intestinal microbiota diversity in CRC patients and healthy individuals based on fecal samples. Note: (**A**) Rarefaction curves depicting intestinal microbiota α-diversity in the Cancer and Normal groups; **B**-**G**: Alpha diversity analysis of intestinal microbiota in the Cancer and Normal groups, with B representing the Chao1 index, C representing the Invsimpson index, D representing the Richness index, E representing the Shannon index, F representing the Simpson index, and G representing the ACE index; (**H**) Beta diversity analysis of intestinal microbiota in the Cancer and Normal groups depicted in a principal coordinate analysis plot; (**I**) Venn diagram illustrating the overlap of OTUs (Operational Taxonomic Units) between the Cancer and Normal groups; lowercase letters (a and a) denote non-significant differences between the two groups in Figure B-G (P > 0.05). The sample size (n) is 10

The study aimed at elucidating the molecular mechanisms through which the intestinal microbiota affects CRC commenced with an analysis of the differences in gut microbiota composition between individuals diagnosed with CRC and those deemed healthy. The investigation obtained CRC-related 16 s rRNA sequencing data set PRJEB6070 from the SRA database and randomly selected 10 samples from both the CRC and healthy control groups. The study first analyzed the richness using an α-rarefaction curve and found that with an increasing percentage of samples, the rate of increase in richness for both the Cancer and Normal groups gradually slowed down and eventually reached saturation (Fig. 1A). This indicates that at the current sequencing depth, the majority of microbial species have been detected. Further evaluation of the species diversity differences between the two groups was performed using alpha diversity analysis, which calculates the species composition within samples based on abundance and richness. Commonly used algorithms include Chao1, Invsimpson, Richness, Shannon, Simpson, and ACE indices. The findings indicated that in comparison to the Cancer group, the Normal group had a noticeable decline in Chao1, Invsimpson, Richness, Shannon, Simpson, and ACE (Fig. 1B-G). Subsequently, the study displayed the beta diversity between samples using PCoA, which is an indicator of the differences in species composition between communities. The results showed some spatial separation between the two groups, particularly in Principal Component 1 and Principal Component 2 (Fig. 1H), indicating distinct differences in the gut microbiota structure of individuals suffering from CRC compared to individuals with no medical issues. Additionally, the study performed Venn analysis on the obtained OTUs from the Cancer and Normal groups. The results revealed that the Cancer group had 78 OTUs different from the Normal group, while the Normal group had 58 OTUs different from the Cancer group (Fig. 1I, Table S4), indicating both commonalities and notable distinctions in the gut microbiota between the Normal and Cancer groups.

These findings demonstrate clear differences in the richness and quantity of gut microbiota between CRC patients and individuals in good health.

### Disparities in the microbial composition of the intestinal flora among individuals with CRC and those without the disease and identification of key species

**Fig. 2:**
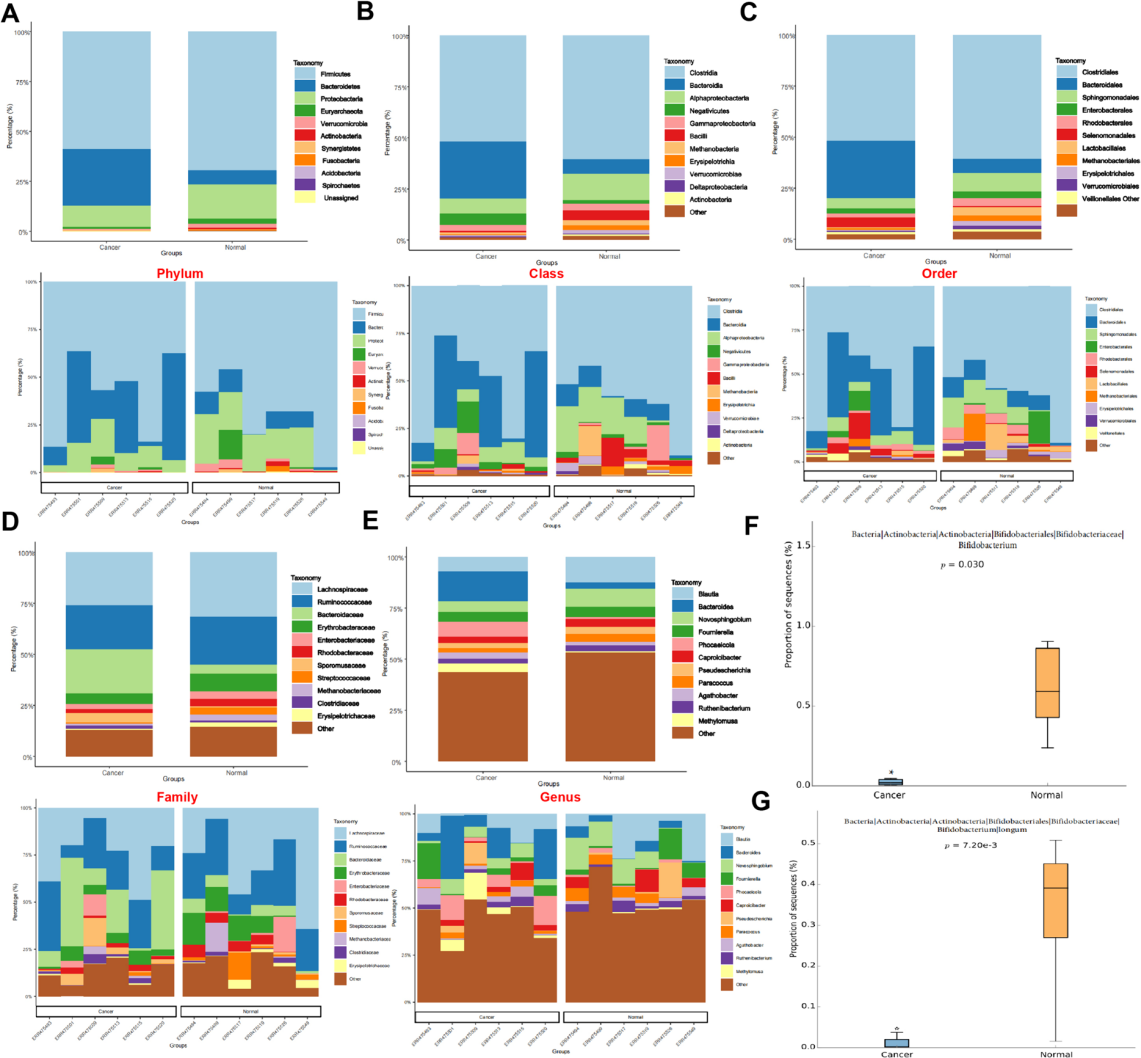
Differential analysis of gut microbial composition at various taxonomic levels between cancer and normal groups. Note: (A) Stacked bar chart depicting the relative abundance of gut microbial species at the phylum level in the Cancer and Normal groups; (**B**) Stacked bar chart illustrating the relative abundance of gut microbial species at the class level in the Cancer and Normal groups; (**C**) Stacked bar chart showing the relative abundance of gut microbial species at the order level in the Cancer and Normal groups; (**D**) Stacked bar chart displaying the relative abundance of gut microbial species at the family level in the Cancer and Normal groups; (**E**) Stacked bar chart presenting the relative abundance of gut microbial species at the genus level in the Cancer and Normal groups; (**F**) Abundance analysis at the genus level of Bifidobacterium in the Cancer group and Normal group; (**G**) Abundance analysis at the species level of Bifidobacterium longum in the Cancer group and Normal group; using Welch's t-test (F-G). Sample size, n = 10. * indicates statistical significance compared to the Normal group (P < 0.05)

To delve deeper into the discrepancies in species composition of gut microbiota among CRC patients and individuals in good health, an examination was carried out across various taxonomic levels including phylum, class, order, family, and genus, with the visualization of findings in a stacked bar chart (Table S5). The results revealed that both groups of microbiota were mainly distributed under the phyla Firmicutes, Bacteroidetes, and Proteobacteria. In contrast to the cancer cohort, the control group exhibited markedly elevated levels of microbiota within Firmicutes and Proteobacteria, whereas the cancer cohort showed notably increased levels of microbiota belonging to Bacteroidetes (Fig. 2A). At the class level, both groups of microbiota were mainly distributed under the classes Clostridia, Bacteroides, and Alphaproteobacteria. When juxtaposed with the cancer group, the group in good health demonstrated notably elevated levels of microbiota in Clostridia and Alphaproteobacteria, while the cancer group displayed significantly elevated levels of microbiota in Bacteroidia (Fig. 2B). At the order level, both groups of microbiota were mainly distributed under the orders Clostridiales, Bacteroidales, and Sphingomonadales. Relative to the cancer group, the group with a healthy status exhibited significantly higher microbiota levels in Clostridiales and Sphingomonadales, while the levels of microbiota in the Bacteroidales order were notably elevated in the group affected by cancer (Fig. 2C). At the family level, both groups of microbiota were mainly distributed under the families Lachnospiraceae, Ruminococcaceae, and Bacteroidaceae. Contrasting with the cancer group, the healthy group displayed a marked increase in the levels of microbiota in Lachnospiraceae, while the cancer group had significantly higher levels of microbiota in Bacteroidaceae (Fig. 2D). At the genus level, both groups of microbiota were mainly distributed under the genera Blautia, Bacteroides, and other genera. In a comparison of the cancer group, the healthy group demonstrated notably elevated levels of microbiota in Blautia, while the group diagnosed with cancer exhibited markedly elevated levels of microbiota in the Bacteroides genus (Fig. 2E).

To further explore the variations in gut microbiota between patients with CRC and individuals in good health, the study identified key species and conducted an analysis of the abundance values of microbiota between the two groups at the genus level. The data analysis and results visualization were conducted through the utilization of STAMP software, employing Welch's t-test for the examination of variances. It was observed that the levels of Bifidobacterium exhibited a significant decrease in the cancer group compared to the healthy group (Fig. 2F). Furthermore, at the species level, an analysis focused on the variation among species was executed, and the results showed that the cancer group manifested a substantial reduction in Bifidobacterium longum abundance in comparison with the healthy group (Fig. 2G).

It is evidenced that there are substantial distinctions in the species makeup of gut microbiota when comparing CRC patients to individuals in good health. Specifically, Bifidobacterium longum may be a key bacterium influencing gut health. This discovery has important implications for a more profound comprehension of the link between CRC and gut microbiota.

### Association between functional distinctions in gut microbiota and CRC development

To further explore the functional variances in gut microbiota among CRC patients and individuals in good health, the study conducted KEGG enrichment analysis using PIParkinsonismUSt2 software, focusing on the genus-level abundance data. Leveraging the STAMP software, the research obtained visual results of the functional composition of gut microbiota. The results showed significant enrichment (P-value < 0.05) in 12 signaling pathways for differentially abundant gut microbiota between the Cancer and Normal groups. These pathways were the Sulfur relay system, Zeatin biosynthesis, One carbon pool by folate, Restriction enzyme, Glutamatergic synapse, Type I diabetes mellitus, Alanine, aspartate and glutamate metabolism, Staphylococcus aureus infection, Chloroalkane and chloroalkene degradation, Butirosin and neomycin biosynthesis, Carbon fixation in photosynthetic organisms, and Histidine metabolism (Figure S1A-B). Particularly, among these pathways, the most significant difference was observed in Alanine, aspartate and glutamate metabolism. The metabolism of Alanine, Aspartate, and Glutamate participates in various central metabolic pathways, such as glycolysis, gluconeogenesis, citric acid cycle, and ammonia metabolism. Earlier investigations have proven that an excess of ammonia metabolism can hinder the multiplication of CRC cells, and this phenomenon is intricately associated with UC metabolism (Zhang et al. 2019), in line with the outcomes of the analysis.

Therefore, the above findings indicate noticeable functional changes in gut microbiota between individuals diagnosed with CRC and those who are considered healthy. These functional changes could potentially contribute to the onset and advancement of CRC by the modulation of the ammonia metabolism pathway.

### Bifidobacterium abundance reduction and its correlation with CRC progression: investigating the research results

**Fig. 3:**
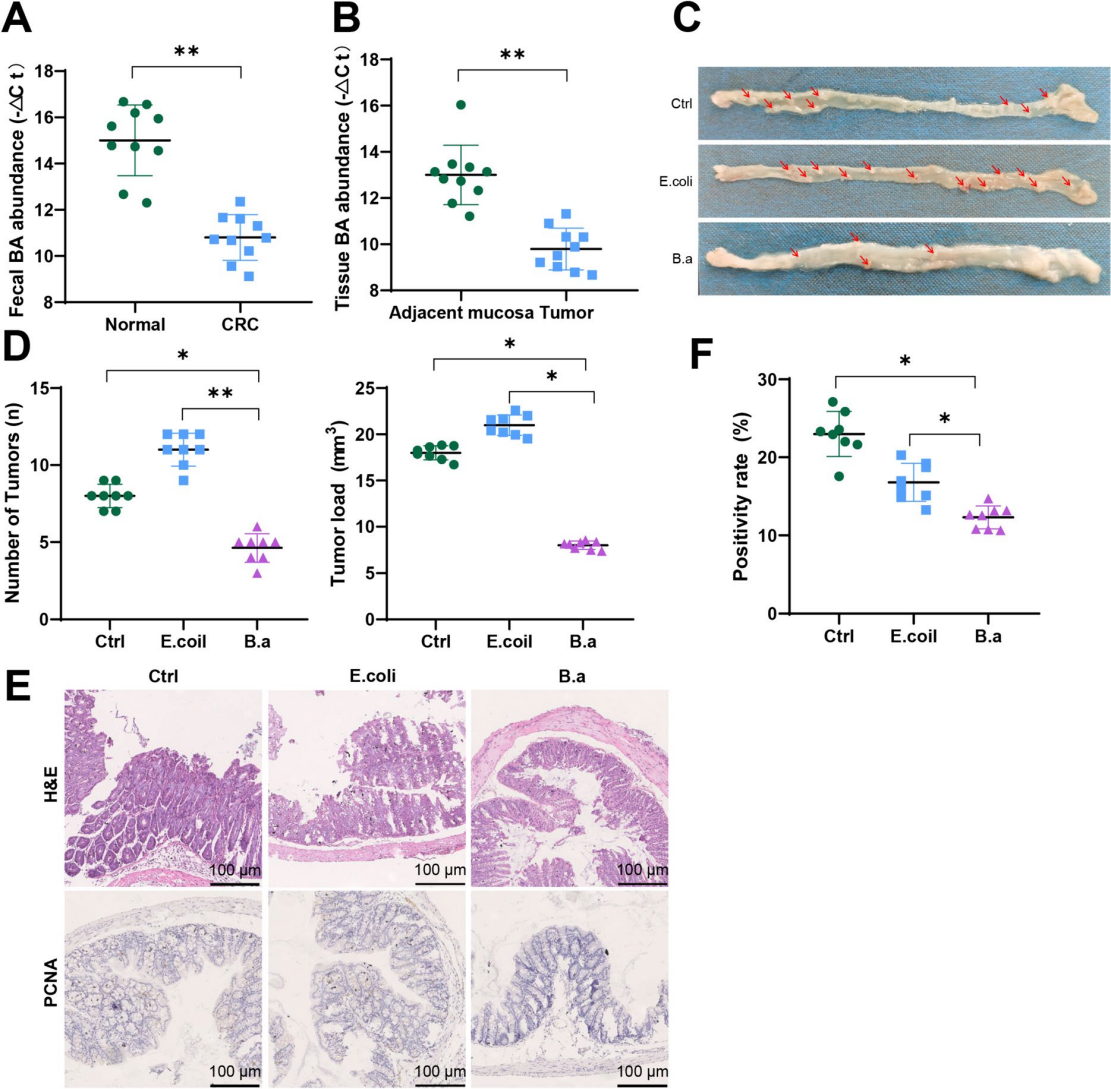
Changes in abundance of Bifidobacterium in CRC patients and the therapeutic effect of Bifidobacterium supplementation on CRC. Note: (**A**) The expression of Bifidobacterium genomic DNA in fecal samples of healthy individuals and CRC patients was studied; (**B**) The expression of Bifidobacterium genomic DNA in tumor tissue and adjacent normal mucosa tissue was studied; (**C**) Representative images of the colon in mice from different treatment groups, with tumor tissue marked in red; (**D**) Quantification of tumor number (left panel) and tumor volume (right panel) in the colon of mice from different treatment groups; (**E**) Pathological changes in mouse tumor tissue were examined using HE staining, and the expression of PCNA in mouse tumor tissue was detected by immunohistochemistry, scale bar = 100 μm; (**F**) Statistics of PCNA immunohistochemical protein positivity. * represents P < 0.05 between the two groups, n = 8 for mouse experiments, n = 10 for clinical samples

To investigate whether B.a has an impact on CRC progression, the research collected clinical samples and initially evaluated the levels of B.a abundance in healthy individuals and CRC patients. The research collected fecal samples from healthy individuals and CRC patients, as well as CRC tissues and adjacent normal mucosal tissues. Genomic DNA from bacterial genomes was extracted from the samples, and the abundance of B.a was assessed utilizing RT-qPCR. There was a marked decline observed in the presence of B.a in the fecal samples of CRC patients as opposed to the healthy subjects, as indicated by the results (Fig. 3A). Furthermore, the concentration of B.a was notably reduced in neoplastic tissue in contrast to neighboring healthy mucosal tissues (Fig. 3B). This finding is consistent with the bioinformatics analysis, suggesting a significant reduction in B.a abundance in CRC patients.

To further investigate the impact of B.a on CRC progression, the research used an AOM/DSS-induced colon tumor model that mimicked chronic colon inflammation. The research administered Escherichia coli, BA, or PBS treatment and recorded the incidence of intestinal tumors. The results indicated a notable reduction in the number and volume of intestinal tumors in the B.a group contrasting with the control group or the E. coli group (Fig. 3C-D). Histopathological changes in the intestines were assessed using H&E staining, and the expression of the tumor proliferation marker, proliferating cell nuclear antigen (PCNA), was assessed using immunohistochemical staining. The results demonstrated a significant improvement in intestinal pathology and a reduction in PCNA expression in the B.a group relative to the control group or the E. coli group (Fig. 3E-F).

In conclusion, the findings above indicate a significant reduction in B.a abundance in patients with CRC. Supplementation of Bifidobacterium can effectively inhibit the progression of CRC in an AOM/DSS-induced colon tumor model.

### Intestinal microbiota regulates bifidobacterium to control polyamine production in patients with CRC and UC

The intestinal microbiota, particularly Bifidobacterium, has been shown in recent studies to have an impact on host urea metabolism. This impact is achieved primarily through two mechanisms: direct breakdown of urea to obtain nitrogen for its own growth and survival and indirect regulation of the activity of UC enzymes in the host liver by producing short-chain fatty acids or other metabolites (Tojo et al. 2014).

**Fig. 4:**
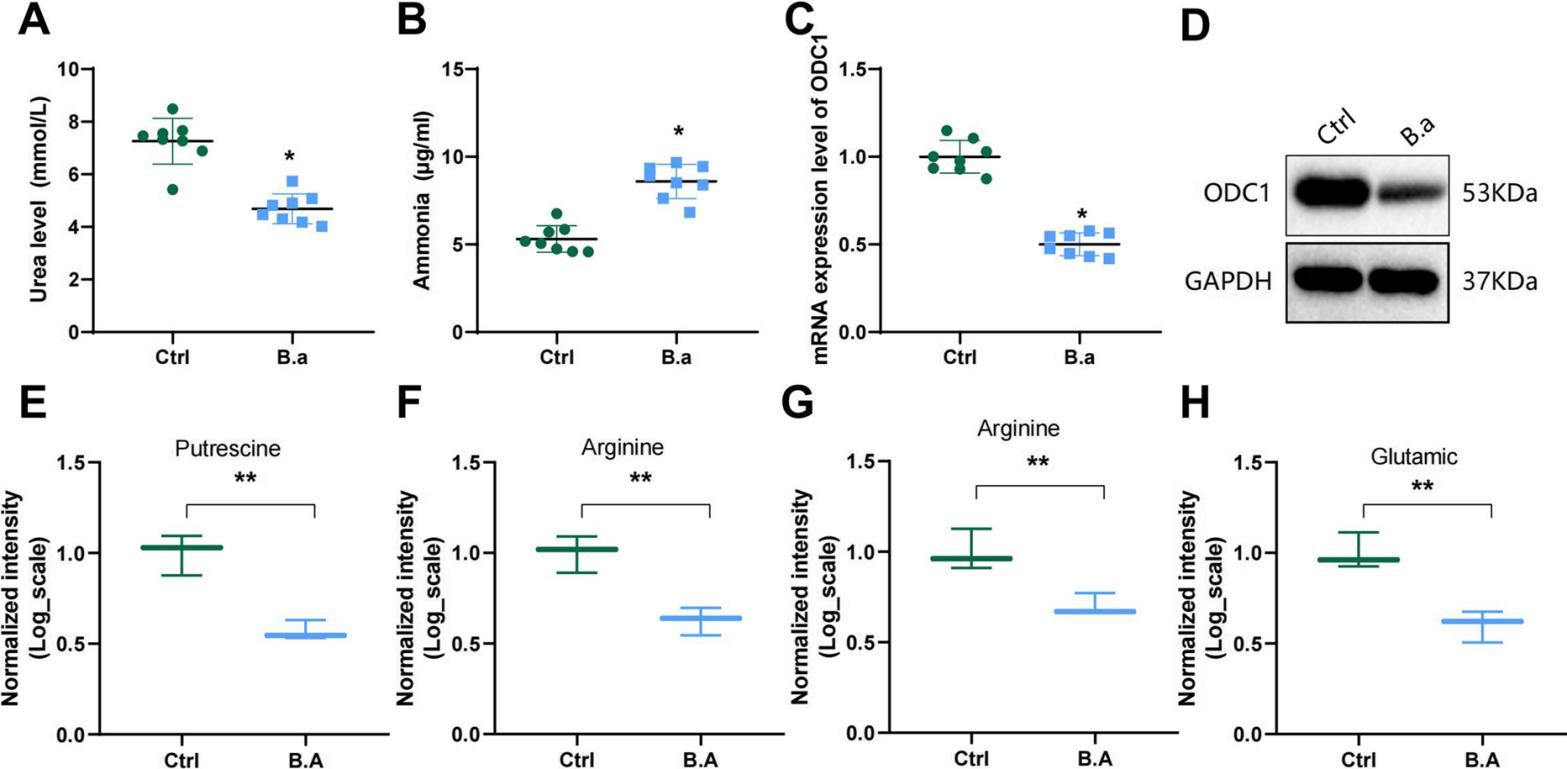
Influence of Bifidobacterium on UC metabolism and related metabolites. Note: (**A**) The content of urea in tumor tissue of mice in different treatment groups was detected using a reagent kit. (**B**) The level of ammonia in tumor tissue of mice in different treatment groups was detected using a reagent kit. (**C**) The mRNA expression of ODC1 in tumor tissue of mice in different treatment groups was detected using RT-qPCR. (**D**) The protein expression of ODC1 in tumor tissue of mice in different treatment groups was detected using Western Blot. (**E**) The metabolic level of putrescine in tumor tissue of mice in different treatment groups was detected using LC–MS/MS. (**F**) The metabolic level of aspartic acid in tumor tissue of mice in different treatment groups was detected using LC–MS/MS. (**G**) The metabolic level of arginine in tumor tissue of mice in different treatment groups was detected using LC–MS/MS. (H) The metabolic level of Glutamate in tumor tissue of mice in different treatment groups was detected using LC–MS/MS. * represents P < 0.05 compared to the Ctrl group; * indicates P < 0.01 compared to the Ctrl group

To examine the regulatory function of Bifidobacterium in the UC in CRC and to explore the complex interactions between the gut microbiota and individuals with CRC, the research established a Bifidobacterium group (B.a group) and a control group (Ctrl group) in a mouse model and measured the levels of urea and ammonia in their intestinal tissues. The B.a group showed notable decreases in urea levels and significant increases in ammonia levels in comparison to the Ctrl group, as highlighted in the study outcomes (Fig. 4A-B).

One intermediate product of the UC is ornithine decarboxylase 1 (ODC1), a specific substrate that is essential for the production of polyamines during tumor growth (Cervelli et al. 2014). Polyamines, such as putrescine, spermidine, and their precursor cadaverine, play a central role in multiple stages of colorectal tumor development. Additionally, changes in polyamine levels are closely correlated with the growth vitality of CRC cells, and alterations in intracellular polyamine content are among the most consistent biochemical changes related to their proliferative capacity (Bachrach 2004).

To further investigate this, RT-qPCR and Western blot assays were carried out to evaluate the ODC1 expression, a key enzyme involved in polyamine metabolism, in mouse tumor tissues. The experimental results illustrated that in relation to the Ctrl group, the B.a group had significantly decreased mRNA and protein expression of ODC1 (Fig. 4C-D). This suggests that supplementation with Bifidobacterium can significantly inhibit urea metabolism and polyamine production. To validate the changes in urea metabolism, the measurement of UC intermediates (such as putrescine, arginine, aspartic acid and glutamate) in tumor tissues was conducted using liquid chromatography-mass spectrometry (LC–MS). A decrease in putrescine, arginine, aspartic acid, and glutamate levels was evident in the B.a group as opposed to the Ctrl group based on the study outcomes (Fig. 4E-H).

Overall, the research highlights the regulatory impact of Bifidobacterium on the UC of CRC patients, and supplementation with Bifidobacterium can inhibit urea metabolism and polyamine production.

### Bifidobacterium regulation of UC metabolism in the progression of CRC

UC is an important metabolic pathway in the body, responsible for the conversion of ammonia to urea to maintain nitrogen metabolism balance (Matsumoto et al. 2019). Recent studies have shown that the gut microbiota can further convert urea into ammonia and other metabolites through their metabolic activities, such as uricase activity. These metabolites may have an impact on the gut environment and epithelial cell function, and the abnormal nitrogen metabolism changes caused by UC may lead to cellular stress, inflammation, and the formation of a pro-cancer environment (Chen et al. 2023b).

**Fig. 5:**
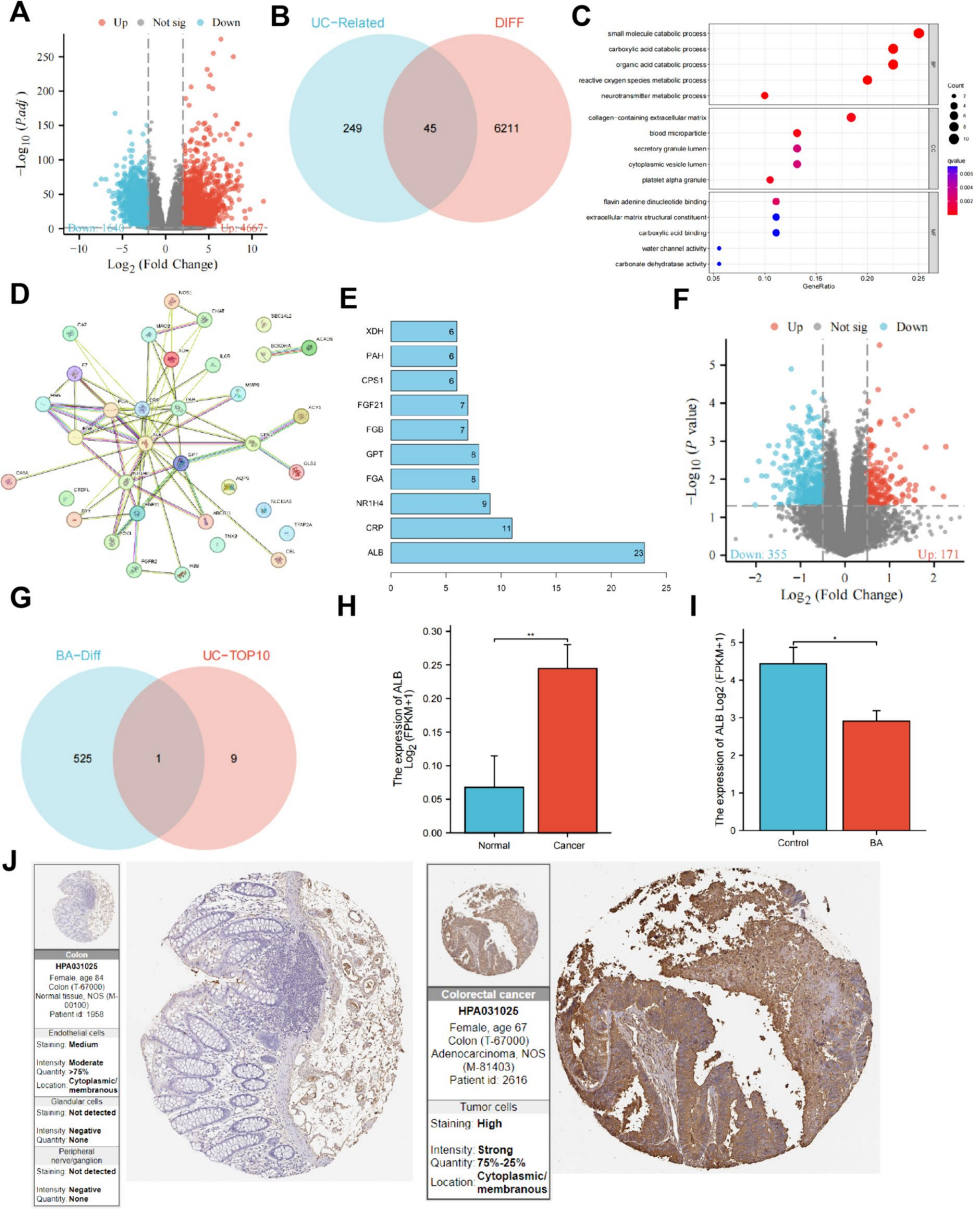
Identification of key genes involved in UC metabolism regulated by Bifidobacterium in CRC through transcriptome sequencing analysis. Note: (**A**) Volcano plot depicting the differential gene expression analysis between healthy individuals (n = 51) and CRC patients (n = 633) from the TCGA database. Red dots represent upregulated genes, blue dots represent downregulated genes, and gray dots represent genes with no significant differential expression. (**B**) Venn diagram showing the intersection between differentially expressed genes in healthy individuals and CRC patients and UC metabolism-related genes. (**C**) Gene Ontology (GO) enrichment analysis results of differentially expressed UC metabolism-related genes. (**D**) Protein–protein interaction network of differentially expressed UC metabolism-related genes, with the edges indicating the existence of interactioaions. (**E**) Bar plot showing the number of connecting nodes in the protein–protein interaction network, where a higher number indicates a higher degree of centrality. (**F**) Volcano plot illustrating the differential gene expression analysis between colon tissues of BA-fed mice and control group mice, with red dots representing upregulated genes, blue dots representing downregulated genes, and gray dots representing genes with no significant differential expression. (**G**) Venn diagram depicting the intersection between BA-associated differentially expressed genes and the top ten genes ranked by core centrality in UC metabolism-related differentially expressed genes in CRC. (**H**) Expression of ALB in healthy individuals and CRC patients, with * representing P < 0.01. (**I**) Gene expression of ALB in the Control group and B.a group, with * representing P < 0.05. (**J**) HPA database analysis of ALB protein expression in normal colon tissue and CRC patient tumor tissue

To investigate the changes in UC metabolism associated with CRC progression, the research first analyzed the transcriptome dataset related to CRC in the TCGA database. The results showed that in CRC patients, 1640 genes were significantly downregulated, while 4667 genes were significantly upregulated, including ALB (Fig. 5A). Subsequently, the research used the GeneCard online database to search for the keyword "urea cycle" and obtained a total of 294 UC metabolism-related genes. The research then intersected these genes with the differentially expressed genes and found that 45 UC metabolism-related genes showed significant differential expression in CRC (Fig. 5B). GO enrichment analysis of the differentially expressed UC metabolism key genes unveiled that these genes were mainly enriched in functional categories such as "small molecule catabolic process," "carboxylic acid catabolic process," and "organic acid catabolic process" (Fig. 5C). The UC plays a vital bridging role in these metabolic processes. To identify genes with significant regulatory roles in CRC, the research developed a network diagram of protein interactions involving the UC metabolism-related genes that showed differential expression and further evaluated the core degree of the genes, selecting the top 10 genes (Fig. 5D-E) based on their core degree rankings.

Subsequently, the research conducted an experiment in which mice were fed B.a and intestinal tissue samples were collected for transcriptome sequencing. Differential analysis of sequencing data revealed that differing from control group of mice, the group fed with B.a displayed 526 differentially expressed genes. Among these genes, 355 were significantly downregulated, and 171 were significantly upregulated (Fig. 5F). Next, the research performed an intersection analysis of the BA-related differentially expressed genes with the top ten genes ranked by the core degree that impacts UC metabolism in CRC and found a common gene, ALB (Fig. 5G). Presentation of ALB expression levels revealed that in CRC patient tumor tissues from the TCGA database, the levels of ALB mRNA and protein were notably upregulated in comparison to those found in normal tissues (Fig. 5H; J). In contrast, sequencing results from mouse intestinal tissues showed a significant downregulation of ALB in the B.a group contrasting with the control group (Fig. 5I). ALB is a human gene that encodes serum albumin, one of the most abundant proteins in human plasma. Serum albumin plays a role as a carrier for ammonia in the extracellular fluid, binding and "storing" ammonia until it is safely excreted or metabolized.

In summary, the research indicates that Bifidobacterium can inhibit UC metabolism in CRC patients by downregulating ALB, thereby reducing ammonia levels in the body.

### Bifidobacterium inhibits CRC cell proliferation by regulating ALB expression

**Fig. 6:**
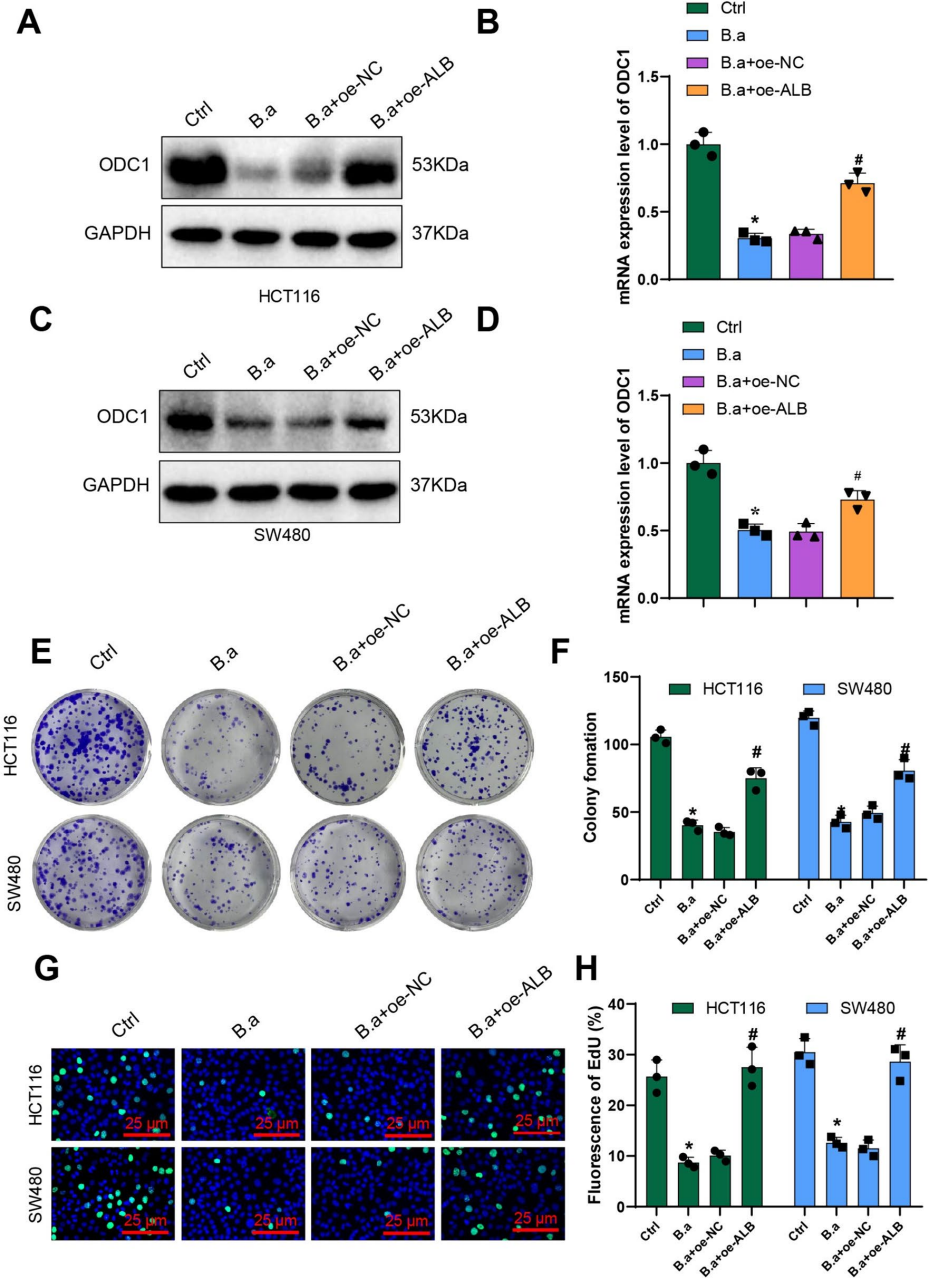
Relationship between Bifidobacterium-mediated CRC cell proliferation and key enzyme ALB involved in UC metabolism. Note: (**A**) Western blot analysis to detect the expression of ODC1 protein in HCT116 cells; (**B**) RT-qPCR analysis to determine the expression of ODC1 mRNA in HCT116 cells; (**C**) Western blot analysis to evaluate the expression of ODC1 protein in SW480 cells; (**D**) RT-qPCR analysis to assess the expression of ODC1 mRNA in SW480 cells; (**E**) Colony formation assay to measure the colony formation ability of HCT116 and SW480 cells after different treatments; (**F**) Statistical analysis of colony formation assay in HCT116 and SW480 cells; (**G**) EdU immunofluorescence assay to investigate the cell proliferation of CRC cells; (**H**) Statistical analysis of EdU immunofluorescence intensity. The mentioned cellular experiments were replicated three times. * indicates significant difference compared to the Ctrl group with P < 0.05; # indicates significant difference compared to the B.a + oe-NC group with P < 0.05

Suppression of the organism UC results in abnormal accumulation of ammonia. Hyperammonemia is not only associated with neurological complications but also with the onset and progression of certain diseases, including CRC (Polletta et al. 2015; Okamoto et al. 2021). In the bioinformatics analysis, the research found that Bifidobacterium may have a regulatory effect on ALB, a key gene in CRC progression. To investigate whether Bifidobacterium can affect CRC cell proliferation by regulating ALB, the research co-cultured CRC cell lines HCT116 and SW480 and overexpressed mouse UC metabolic key factor ALB using lentiviral transduction. The mRNA and protein expression levels of ODC1 in each group of cells were detected by RT-qPCR and WB experiments. There was a significant reduction in the mRNA and protein expressions of ODC1 in the B.a group in comparison with the Ctrl group. When compared to the B.a + oe-NC group, a marked rise in ODC1 mRNA and protein expression levels was observed in the B.a + oe-ALB group (Fig. 6A-D). Furthermore, colony formation experiments were carried out to evaluate the colony formation capacity of CRC cells. A marked decrease in the ability of colony formation was observed in the B.a group in contrast to the control group. When compared to the B.a + oe-NC group, a substantial increase in colony formation ability was noted in the B.a + oe-ALB group (Fig. 6E-F). Cell proliferation was assessed using the Edu fluorescence assay, and colony formation ability was examined through colony formation experiments. In comparison with the B.a + oe-NC group, the results revealed that the B.a + oe-ALB group had significantly increased CRC cell proliferation and colony formation ability (Fig. 6G-H). These findings indicate that Bifidobacterium inhibits CRC cell proliferation by downregulating ALB expression.

### ALB inhibitor bifidobacterium promotes the repair of mitochondrial function in CRC cells by reducing oxidative stress

ALB is an inhibitor of ALB/Ba that promotes the repair of mitochondrial function in CRC cells by reducing oxidative stress (Angelova et al. 2022; Davuluri et al. 2016). Mitochondria, which play a crucial role in a range of events from embryonic development to controlling cell death, are also involved in cancer growth and progression (Tailor et al. 2014). In order to investigate the impact of ALB expression changes on mitochondrial function in CRC cells, the research examined the changes in mitochondrial function-related indicators in CRC cell lines overexpressing ALB.

**Fig. 7:**
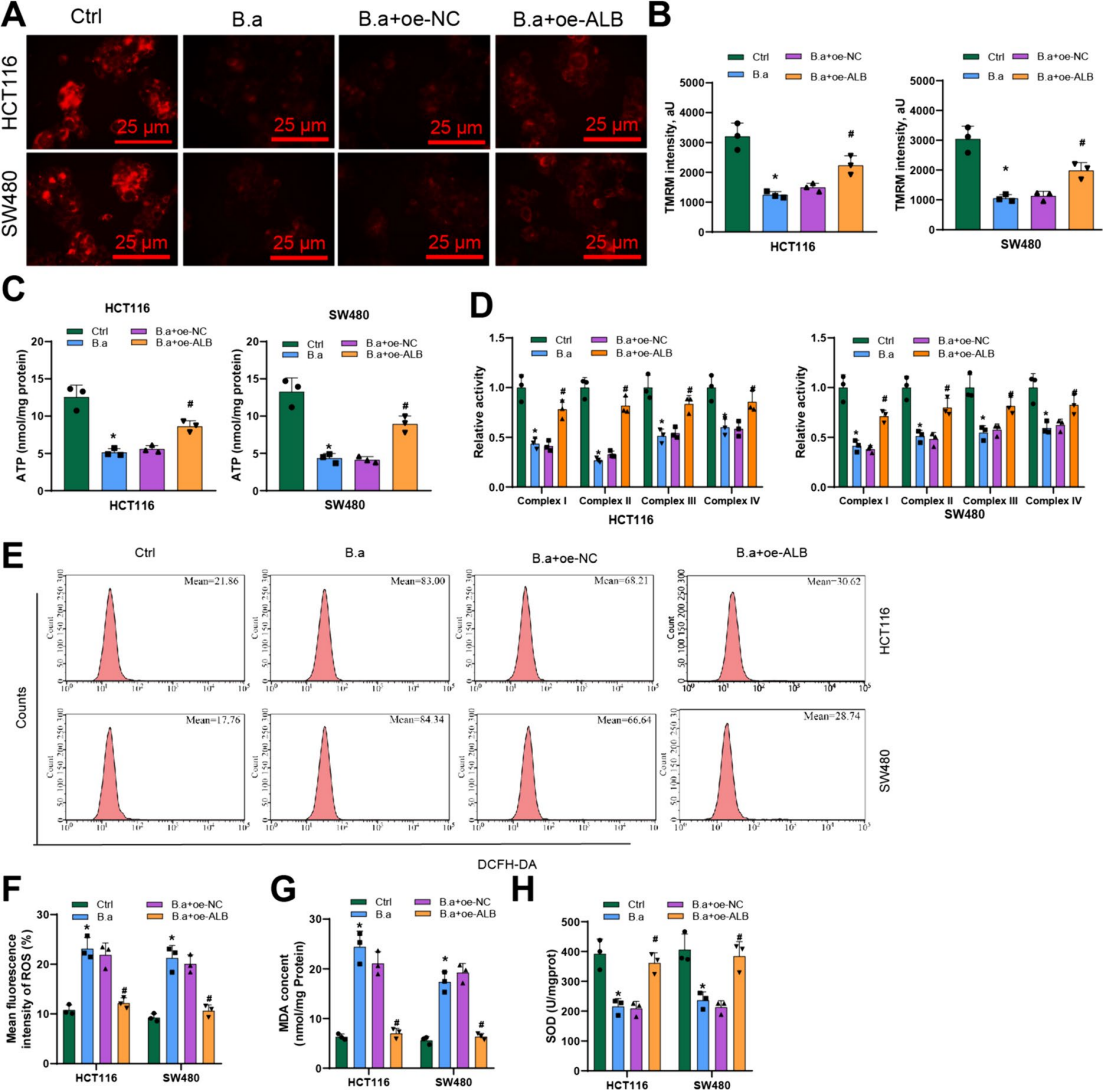
Inhibition of ALB by Bifidobacterium is associated with mitochondrial autophagy. Note: (**A**) TMRM fluorescence assay to investigate the change in Δψm in CRC cells treated with different conditions; (**B**) Quantitative bar graph depicting the change in Δψm; (**C**) ATP assay kit to measure the ATP levels in CRC cells treated with different conditions; (**D**) Assay kit to evaluate the activity of mitochondrial complexes I, II, III, and IV in CRC cells; (**E**) Detecting the ROS levels in CRC cells after treatment with ammonia using flow cytometry; (**F**) Quantitative statistical graph of average fluorescence intensity for ROS detection; (**G**) ELISA assay kit to measure the MDA levels in CRC cells treated with different conditions; (**H**) ELISA assay kit to determine the SOD levels in CRC cells treated with different conditions. The mentioned cellular experiments were replicated three times. * indicates significant difference compared to the control group with P < 0.05; # indicates significant difference compared to the B.a + oe-NC group with P < 0.05

The Δψm serves as common marker to assess the well-being and operation of mitochondria. The research evaluated Δψm in CRC cells using the TMRM staining method. A marked decline in Δψm was observed in the B.a group when contrasted with the Ctrl group, according to the results. Furthermore, the B.a + oe-ALB group exhibited a significant increase in Δψm compared to the B.a + oe-NC group (Fig. 7A-B). The research further assessed the functioning of mitochondrial complexes I, II, III, and IV using commercial assay kits and also assessed the ATP levels in CRC cells. The results demonstrated a marked downturn in the activity of mitochondrial complexes and ATP levels in the B.a group when juxtaposed with the Ctrl group. In opposition, the B.a + oe-ALB experimental set demonstrated a marked upsurge in mitochondrial complex activity and ATP levels relative to the B.a + oe-NC group (Fig. 7C-D).

Elevated ammonia levels can enhance the formation of ROS. Subsequently, the research investigated the changes in oxidative stress levels in CRC cell lines by using flow cytometry to detect ROS production within the cells. The production of ROS was examined by the formation of fluorescent dichlorofluorescein (DCF). The results revealed a marked uptick in ROS levels in the B.a group relative to the Ctrl group. Conversely, the B.a + oe-ALB group displayed a notable reduction in ROS production in CRC cells in comparison to the B.a + oe-NC group (Fig. 7E-F). The study measured the levels of MDA and SOD in cells using ELISA assay kits. The findings indicated a noteworthy elevation in MDA concentrations and a substantial decline in SOD concentrations in the B.a group compared to the Ctrl group. On the contrary, the B.a + oe-ALB group exhibited a noteworthy decrease in MDA levels and a substantial increase in SOD levels in CRC cells as opposed to the B.a + oe-NC group (Fig. 7G-H).

The above findings indicate that Bifidobacterium can induce oxidative stress in CRC cells by inhibiting ALB, leading to mitochondrial dysfunction.

### Inhibition of CRC progression by BA-induced *ammonia* accumulation via downregulation of ALB gene

**Fig. 8:**
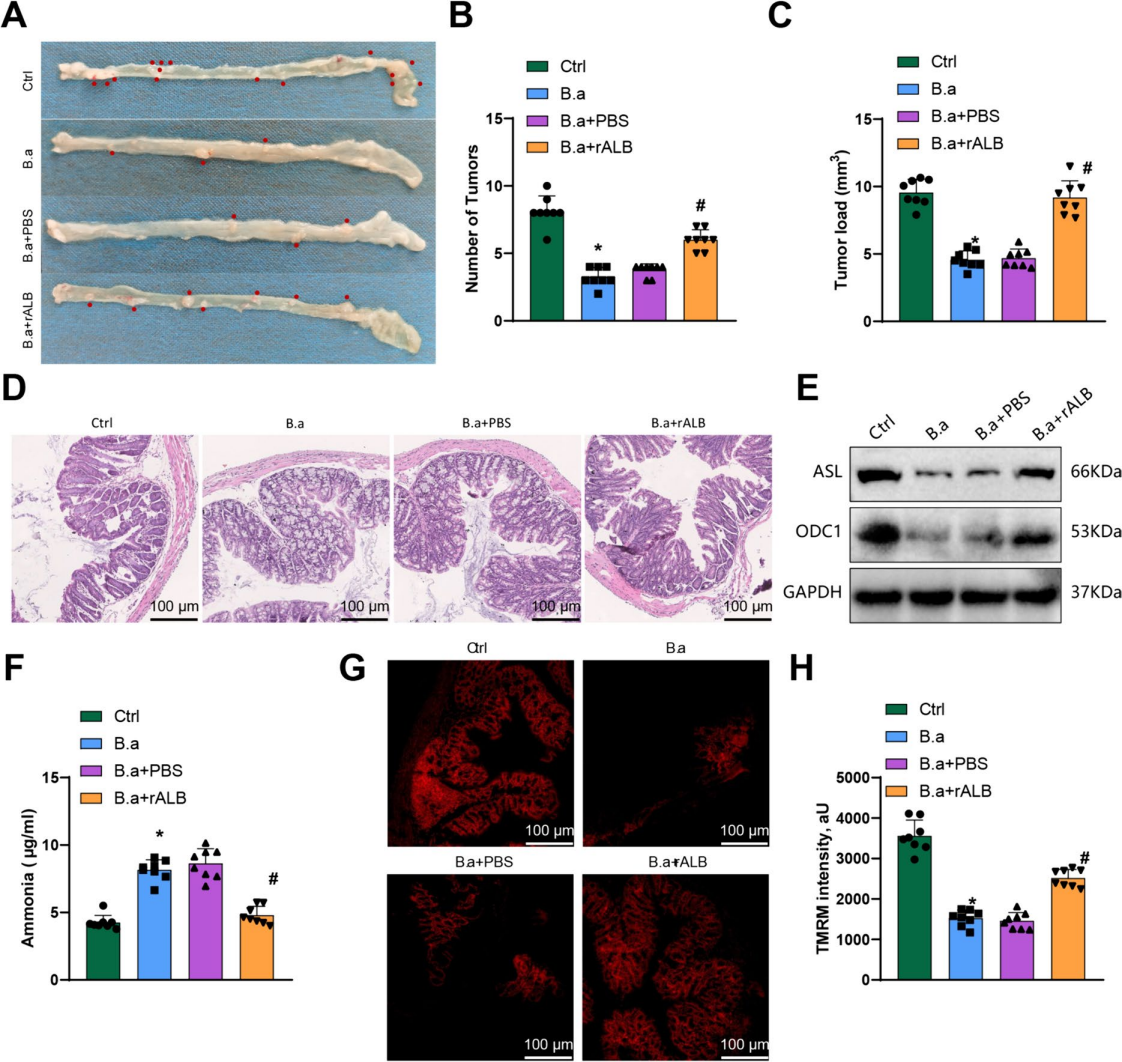
Bifidobacterium induces mitochondrial dysfunction by downregulating the ALB gene, resulting in ammonia accumulation and subsequently inhibiting CRC progression. Note: (**A**) Representative images of mouse colons from different treatment groups, with red indicating tumor tissue; (**B**) Statistical graph showing the number of colon tumors in different treatment groups; (**C**) Statistical graph showing the tumor volume in different treatment groups; (**D**) HE staining showing the histopathological changes in mouse tumor tissues, scale bar = 100 μm; (**E**) Western blot analysis showing the expression of ALB and ODC1 proteins in tumor tissues; (**F**) Reagent kit used to measure ammonia levels in tumor tissues of different treatment groups; (**G**) TMRM fluorescence assay detecting changes in Δψm in CRC cells from different treatment groups; (**H**) Statistical graph showing the changes in Δψm in CRC cells from different treatment groups measured using the TMRM fluorescence assay. * indicates P < 0.05 compared to the Ctrl group; # indicates P < 0.05 compared to the B.a + PBS group. Mouse experiments, n = 8

To further investigate the specific mechanism by which Bifidobacterium regulates CRC progression, the research employed a lentiviral injection technique to overexpress the key gene ALB in the mouse model while supplementing with Bifidobacterium. After completion of the treatment, the research collected intestinal and tumor tissues. Analysis of the tumor tissues unveiled that in relation to the Ctrl group, the number and volume of intestinal tumors were significantly reduced in the B.a group (Fig. 8A-C). Furthermore, compared to the B.a + PBS group, the B.a + rALB group showed a remarkable increase in the number and size of intestinal tumors (Fig. 8A-C). Subsequently, the research investigated the pathological changes in the intestines using HE staining. The outcomes pointed out that in relation to the Ctrl group, the severity of lesions was alleviated in the B.a group. In contrast, as opposed to the B.a + PBS group, the severity of lesions was exacerbated in the B.a + rALB group (Fig. 8D).

To determine the mRNA and protein expression levels of ALB and ODC1 in each group of mice, the research performed RT-qPCR and WB experiments. The data illustrated that in comparison to the Ctrl group, the expression levels of ALB and ODC1 mRNA and protein experienced a notable decline in the B.a group. Conversely, relative to the B.a + PBS group, the B.a + rALB group exhibited a marked increase in the expression levels of ALB and ODC1 mRNA and protein (Fig. 8E).

The study measured the ammonia levels in the tumor tissues of each group using an ammonia assay kit. A substantial rise in ammonia levels within the tumor tissues was observed in the B.a group when compared to the Ctrl group, as per the results. In contrast, as opposed to the B.a + PBS group, the B.a + rALB group displayed a considerable downturn in ammonia levels (Fig. 8F). To investigate the Δψm in the tumor tissues, the research performed TMRM staining. A considerable downturn in Δψm was evident in the tumor tissues of the B.a group as compared to the Ctrl group based on the results. Conversely, the Δψm displayed a significant increase in the B.a + rALB group as opposed to the B.a + PBS group (Fig. 8G-H).

Taken together, these findings suggest that Bifidobacterium can induce ammonia accumulation through the downregulation of the ALB gene, leading to mitochondrial dysfunction and inhibition of CRC progression.

## Discussion

This study utilized bioinformatics analysis and experimental validation to uncover the mechanism by which Bifidobacterium regulates CRC through UC modulation. The UC plays a crucial role in cellular metabolism, and Bifidobacterium can influence CRC cell proliferation by regulating the key gene, ALB, involved in this cycle (Makker et al. 2022; Tang et al. 2023; Ribas et al. 2022). This finding, consistent with previous studies, supports the significant role of the UC in CRC and further reveals the regulatory mechanism of gut microbiota on this cycle (Huang et al. 2023).

In comparison to prior research, one innovative aspect of this study is the unveiling of the regulatory role of Bifidobacterium in CRC. Prior studies were chiefly concerned with the abundance changes of certain gut microbiota in CRC, while this study delved deeper into the key genes involved in UC metabolism, revealing the specific mechanisms by which Bifidobacterium regulates CRC progression. These findings offer novel viewpoints and aims for the prevention and therapy of CRC.

Although previous research has shown the significant role of the UC in the emergence and progression of CRC, the understanding of its regulatory mechanisms remains incomplete (Kobayashi et al. 2020; Li et al. 2022; Quaglio et al. 2022). This study, through comprehensive analysis, experimental validation, and in vitro and in vivo studies, first reveals how Bifidobacterium affects CRC cell proliferation and mitochondrial function by modulating the UC. This finding provides new clues for further exploring the regulatory mechanisms of the UC and important guidance for finding new therapeutic strategies and targeted drugs.

Furthermore, this study further verifies the significant decrease in Bifidobacterium abundance in CRC patients, consistent with previous research. However, despite numerous studies on the relationship between gut microbiota and tumors, the specific mechanisms by which Bifidobacterium contributes to CRC occurrence and development still hold many mysteries (Chen et al. 2023b; Li et al. 2021b; Qin et al. 2024).

Based on the experiments conducted, the research can tentatively draw the following conclusions: in CRC patients, the abundance of Bifidobacterium is significantly reduced. The supplementation of Bifidobacterium treatment downregulates the key gene ALB involved in UC metabolism, inhibiting the UC process in CRC patients. This further leads to the accumulation of ammonia, inducing mitochondrial dysfunction in CRC cells and ultimately inhibiting CRC progression. This finding provides a reference for a deeper understanding of the impact of gut microbiota on CRC and also offers new avenues for the development and exploration of future CRC treatment methods. However, the specific molecular mechanisms by which Bifidobacterium influences CRC progression still require further elucidation. Additionally, the research need to further investigate other pathways through which Bifidobacterium affects CRC progression.

The results of this study have significant clinical implications. Firstly, the research discovered that Bifidobacterium modulates UC and impacts mitochondrial function in CRC cells, providing new insights for the development of therapeutic strategies targeting UC. Secondly, in line with existing research, the abundance of Bifidobacterium may serve as a prognostic indicator or evaluation marker for treatment efficacy in CRC (Sakakida et al. 2022). Moreover, this study offers a fresh perspective on the relationship between gut microbiota and tumors, aiding the understanding of the mechanisms underlying tumorigenesis.

While this study has yielded some meaningful findings, there are several limitations that need to be addressed. Firstly, despite our attempts to utilize the LDA Effect Size (LEfSe) analysis to identify significant differences in microbial populations, no significant results were obtained. Secondly, as the experimental data in this study primarily stemmed from a mouse model, further validation is required to assess the translatability of the results to humans. Additionally, although we conducted preliminary exploration into the regulatory mechanisms of UC, further validation of the related molecular mechanisms through various approaches is necessary. The COVID-19 pandemic significantly impacted our sample collection efforts, reducing not only the sample size but also increasing the difficulty in acquiring resources. Moreover, the implemented preventive measures resulted in reduced laboratory personnel density and shortened experimental operation times. The inclusion of only 10 samples each from normal individuals and colon cancer patients, both tissue and fecal samples, limited our statistical analysis capabilities and may have reduced the generalizability and applicability of the study results. However, by leveraging high-throughput sequencing technologies and comprehensive multi-database analyses, we have strived to enhance the data quality and analytical depth of the study. Furthermore, the use of the AOM/DSS mouse model and cell experiments has provided additional biological evidence to support our conclusions.

## Conclusion

Stemming from the outcomes of this research, future research can expand on the interactions between Bifidobacterium and other gut microbiota, as well as their regulatory mechanisms in UC. Additionally, increasing the sample size will enable further verification of the associations between changes in Bifidobacterium abundance and prognosis in CRC. Simultaneously, an in-depth exploration of the regulatory mechanisms underlying UC will facilitate the discovery of potential targeted therapeutic strategies. Lastly, integrating the discoveries of this study with clinical practice will enhance the development of more effective treatment approaches and personalized therapy plans for CRC.

## Supplementary Information

Below is the link to the electronic supplementary material.

Supplementary file1 (JPG 1263 KB)

Supplementary file2 (DOCX 14 KB)

Supplementary file3 (DOCX 12 KB)

Supplementary file4 (DOCX 13 KB)

Supplementary file5 (XLSX 32 KB)

Supplementary file6 (XLSX 75 KB)

## Data Availability

No datasets were generated or analysed during the current study.
