# Supplementary figures and images for: Novel therapeutic targets: bifidobacterium-mediated urea cycle regulation in colorectal cancer
Source: Cell Biol Toxicol. 2024 Aug 3;40(1):64. doi: 10.1007/s10565-024-09889-y (PMC11297826; doi:10.1007/s10565-024-09889-y)

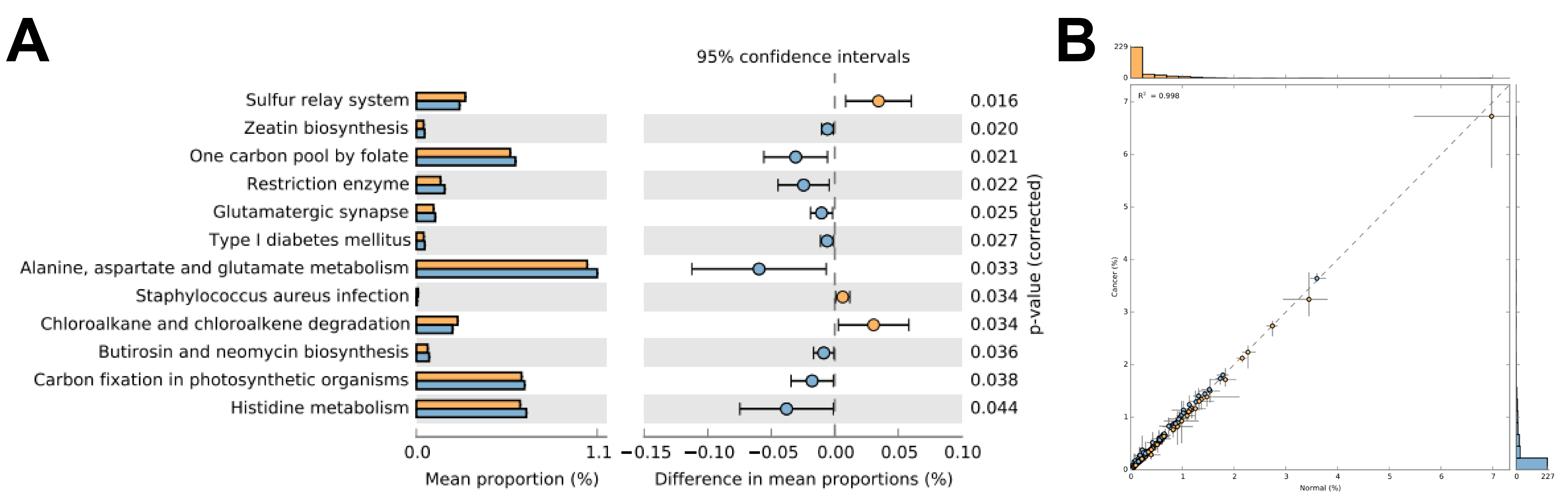

Supplement: Supplementary file 1 — Supplementary file1 (JPG 1263 KB) [file 10565_2024_9889_MOESM1_ESM.jpg]
