# Supplementary material for: Novel therapeutic targets: bifidobacterium-mediated urea cycle regulation in colorectal cancer
Source: Cell Biol Toxicol. 2024 Aug 3;40(1):64. doi: 10.1007/s10565-024-09889-y (PMC11297826; doi:10.1007/s10565-024-09889-y)
Supplement: Supplementary file 2 — Supplementary file2 (DOCX 14 KB) [file 10565_2024_9889_MOESM2_ESM.docx]

**Table S1. Statistical information on the characteristics of patients in the Control and BA groups.**

| **Characteristic Item** | **Control** | **BA** |
| --- | --- | --- |
| Gender |  |  |
| Male | 6 | 5 |
| Female | 4 | 5 |
| Age | 51.6 | 54.8 |
| Disease Stage |  |  |
| I/II | 5 |  |
| Ⅲ/Ⅳ | 5 |  |
| Current Treatment |  |  |
| Surgery + Radiotherapy | 3 |  |
| Surgery | 5 |  |
| Radiotherapy | 2 |  |
| Comorbidities |  |  |
| No metastasis | 7 |  |
| Liver metastasis only | 1 |  |
| Lung metastasis only | 1 |  |
| Both liver and lung metastasis | 1 |  |
| Anatomical Location of Tissue Collection |  |  |
| Rectum | 3 |  |
| Sigmoid colon | 3 |  |
| Transverse colon | 4 |  |
