# Supplementary material for: Novel therapeutic targets: bifidobacterium-mediated urea cycle regulation in colorectal cancer
Source: Cell Biol Toxicol. 2024 Aug 3;40(1):64. doi: 10.1007/s10565-024-09889-y (PMC11297826; doi:10.1007/s10565-024-09889-y)
Supplement: Supplementary file 3 — Supplementary file3 (DOCX 12 KB) [file 10565_2024_9889_MOESM3_ESM.docx]

**Table S2. Primer sequence of RT-qPCR**

| **Genes** | **Sequences (5’-3’)** |
| --- | --- |
| *universal Eubacteria 16s* | F: CGGCAACGAGCGCAACCC |
|  | R: CCATTGTAGCACGTGTGTAGCC |
| *B. adolescenti*s | F: CTCCGCCGCTGATCCGGAAGTCG |
|  | R: AACCAACTCGGCGATGTGGACGACA |
| PGT | F: ATCCCCAAAGCACCTGGTTT |
|  | R: AGAGGCCAAGATAGTCCTGGTAA |
| ODC1 (Mouse) | F: TCCTTGATGAAGGCTTTACTGC |
|  | R: ACATAGAACGCATCCTTATCGTC |
| GAPDH (mouse) | F: AGGTCGGTGTGAACGGATTTG |
|  | R: GGGGTCGTTGATGGCAACA |
