# Supplementary material for: Novel therapeutic targets: bifidobacterium-mediated urea cycle regulation in colorectal cancer
Source: Cell Biol Toxicol. 2024 Aug 3;40(1):64. doi: 10.1007/s10565-024-09889-y (PMC11297826; doi:10.1007/s10565-024-09889-y)
Supplement: Supplementary file 4 — Supplementary file4 (DOCX 13 KB) [file 10565_2024_9889_MOESM4_ESM.docx]

**Table S3.** **Antibody information of Western blot**

| **Target** | **Manufacturer** | **CAS** | **Dilution ratio** |  |
| --- | --- | --- | --- | --- |
| ODC1 (Mouse) | | Thermo Fisher | PA5-21362 | 1:500 |
| ALB (Mouse) | | Thermo Fisher | MA5-32531 | 1:5000 |
| GAPDH (Mouse) | | Thermo Fisher | MA1-16757 | 1:1000 |
